# Postpartum Hormonal Contraceptive Use and Risk of Depression

**DOI:** 10.1001/jamanetworkopen.2025.2474

**Published:** 2025-03-31

**Authors:** Søren Vinther Larsen, Brice Ozenne, Anders Pretzmann Mikkelsen, Xiaoqin Liu, Kathrine Bang Madsen, Trine Munk-Olsen, Øjvind Lidegaard, Vibe Gedsø Frokjaer

**Affiliations:** 1 Neurobiology Research Unit, Copenhagen University Hospital–Rigshospitalet, Copenhagen, Denmark; 2 Department of Clinical Medicine, University of Copenhagen, Copenhagen, Denmark; 3 Department of Public Health, Section of Biostatistics, University of Copenhagen, Copenhagen, Denmark; 4 Department of Gynaecology, Fertility and Obstetrics, Juliane Marie Centre, Copenhagen University Hospital–Rigshospitalet, Copenhagen, Denmark; 5 Department of Gynaecology and Obstetrics, Copenhagen University Hospital–Herlev and Gentofte Hospital, Herlev, Denmark; 6 NCRR-National Center for Register-Based Research, Aarhus University, Aarhus, Denmark; 7 Department of Clinical Research, University of Southern Denmark, Odense, Denmark; 8 Psychiatric Center Copenhagen, Mental Health Services in the Capital Region of Denmark, Copenhagen, Denmark

## Abstract

**Importance:**

Hormonal contraceptive (HC) use is associated with depression. It is, however, unknown whether this is also true in the postpartum period when women have a heightened depression risk and are routinely offered HC treatment.

**Objective:**

To examine whether HC initiation post partum is associated with the development of depression within 12 months post partum.

**Design, Setting, and Participants:**

A population-based cohort study based on nationwide Danish register data was conducted including all primiparous women who gave birth from January 1, 1997, through December 31, 2022. Women were excluded if they had depression within 24 months before delivery, multiple births or stillbirth, or a diagnosis of breast cancer or liver tumor. Data analysis was conducted between March 20, 2023, and January 17, 2025.

**Exposure:**

Hormonal contraceptive initiation within 12 months post partum was treated as a time-varying exposure. Hormonal contraceptive types were categorized as combined oral contraceptives (COCs), combined nonoral contraceptives (CNOCs), progestogen-only pills (POPs), and progestogen-only nonoral contraceptives (PNOCs).

**Main Outcomes and Measures:**

Depression within 12 months post partum, defined as filling an antidepressant prescription or receiving a hospital diagnosis of depression, was the main outcome. Adjusted hazard ratios (AHRs) and average absolute risks of depression within 12 months post partum were estimated using Cox proportional hazards regression and a G-formula estimator.

**Results:**

Of 610 038 first-time mothers, 248 274 (40.7%) initiated HCs within 12 months post partum (mean [SD] age, 27.6 [4.3] years for HC users vs 29.6 [4.8] years for nonusers). Hormonal contraceptive initiation was associated with subsequent depression, with an AHR of 1.49 (95% CI, 1.42-1.56) compared with no use, resulting in an increase in the 12-month absolute risk from 1.36% (95% CI, 1.32%-1.39%) to 1.54% (95% CI, 1.50%-1.57%). The AHR for COC was 1.72 (95% CI, 1.63-1.82); CNOC, 1.97 (95% CI, 1.64-2.36); and PNOC, 1.40 (95% CI, 1.25-1.56). Progestogen-only pill exposure was associated with an instantaneously reduced risk in the early study period, but it was increased late post partum. The earlier COCs were initiated post partum the higher the associated rate ratio of depression.

**Conclusions and Relevance:**

In this cohort study, HC initiation post partum was associated with an instantaneous increased risk of developing depression. The associated risk was higher the earlier it was initiated post partum, at least for COC. This finding raises the issue of whether the incidence of depression post partum is increased by routine HC initiation after childbirth.

## Key Points

**Question:**

Is hormonal contraceptive (HC) initiation post partum associated with the risk of developing depression within 12 months after delivery?

**Findings:**

In this cohort study of 610 038 first-time mothers, HC initiation post partum was associated with a 1.49 times higher instantaneous risk of depression compared with no HC use, and this was consistent across HC types, except for progestogen-only pills, for which it was initially reduced and subsequently increased late post partum. The earlier combined oral contraceptive use was initiated post partum the higher the associated rate ratio of depression.

**Meaning:**

These findings suggest that starting HC post partum is associated with an increased risk of developing depression post partum.

## Teaser

This cohort study examines the risk of postpartum depression in women in Denmark who receive hormonal contraceptives within the first year after delivery.

## Introduction

Hormonal contraceptive (HC) initiation has been associated with an increased risk of developing depression.^1,2,3^ It is unclear whether this also applies in the postpartum period,^4^ when women are already at a heightened risk of developing depression.^5^ In Denmark, as many as 40% of mothers initiate HC methods within the first year after delivery and throughout the past 20 years they have started at a shorter and shorter time interval after delivery.^6^ This raises the issue of whether the routine practice of HC initiation in the postpartum period inflates the already heightened risk of depression. Only a few studies have previously addressed this and found conflicting results; however, they were limited by lack of generalizability, insufficient follow-up time, and insufficient accounting for potential confounders.^4^

Herein, we use the Danish national health registers to investigate HC initiation post partum and depression risk in a large, unselected population with a 1-year follow-up time while accounting for various potential confounders, such as medical indications for HC use. Specifically, the objective is to examine whether HC initiation post partum is associated with an increased risk of depression in the postpartum period compared with no HC exposure and whether it depends on age, HC type, and timing of initiation post partum.

## Methods

### Study Design

This population-based cohort study used health care data from the registers listed in eTable 1 in Supplement 1. Data were linked via the unique personal identification number given to Danish residents at birth or immigration. The data were provided by the Danish e-Health Authority and approval was obtained from the Regional Data Health Board Privacy. No ethical approval or informed consent is required for register-based studies in Denmark. The study was reported according to the Strengthening the Reporting of Observational Studies in Epidemiology (STROBE) reporting guideline.^7^

### Study Population

The source population included all primiparous women living in Denmark for at least 24 months before giving birth between January 1, 1997, and December 31, 2022, identified from the Danish Civil Registration System and the Medical Birth Registry.^8,9^ We excluded women who had received a depression diagnosis or filled a prescription for antidepressant medication within 24 months before delivery to ensure only new cases of depression onset were included, women who had a multiple birth or stillbirth,^10,11^ and women who had a contraindication for any HC type, such as a prior diagnosis of breast cancer or a liver tumor (eFigure 1 in Supplement 1).^12^

### Exposure

Hormonal contraceptive use post partum was treated as a time-varying exposure such that all women contributed to nonexposed time until the day they filled an HC prescription after giving birth and thereafter contributed to the exposed time for the rest of the follow-up period. The exposure was differentiated by the following HC types: combined oral contraceptives (COCs), combined nonoral contraceptives (CNOCs) (patch and vaginal ring), progestogen-only pills (POPs), and progestogen-only nonoral contraceptives (PNOCs) (implant, depot injection, and levonorgestrel-releasing intrauterine system [LNG-IUS]).

### Outcome

The outcome of interest was depression within 12 months after delivery. Depression was defined as filling a prescription for antidepressant medication identified in the National Prescription Registry (Anatomical Therapeutic Chemical codes with initial N06A identifier)^13^ or receiving a hospital discharge diagnosis of depression identified in the National Patient Register (*International Statistical Classification of Diseases and Related Health Problems, Tenth Revision* codes F32-34, F38, F39, and F530).^14^

### Covariates

The following covariates measured at the time of delivery were included in the analyses: maternal age (<20, 20-29, and ≥30 years); highest educational level (<high school, high school or vocational education, or ≥bachelor degree); civil status (married/registered with a partner or not); history of any other mental disorder, including previous use of psychoactive drugs; parental history of mental disorders; medical indications for HC use, including polycystic ovary syndrome, endometriosis, premenstrual syndrome, dysmenorrhea, heavy menstrual bleeding, hirsutism, and acne; in vitro fertilization treatment; preterm birth; instrument-assisted or cesarean delivery; preeclampsia or eclampsia; and pregestational or gestational diabetes. We also controlled for period effects by including calendar year in 5-year bands.

### Statistical Analysis

The mothers were followed up from the day of delivery until 12 months post partum, the development of depression, emigration, death, or the end of the study period, whichever came first. Death being very rare (<0.01%), we neglected the hazard of death when computing risks and average risks. We used Cox proportional hazards regression models adjusted for the listed covariates to estimate hazard ratios (HRs) for developing depression in the postpartum period between HC exposed vs nonexposed individuals. To examine whether the association differed by HC type and age group, we further analyzed the association in 2 Cox proportional hazards regression models: 1 stratified by HC type and another stratified by age group.^15^ We also quantified the associated exposure effect on the risk scale by estimating the average estimated risk of developing depression in the postpartum period under the observed HC use as well as in the hypothetical situation where no individual began HC treatment (eMethods 1 and eFigure 2 in Supplement 1 provide details). In brief, we fitted an additional Cox model^15^ to estimate the hazard of starting HC methods and plugged the estimated transition hazards between states in the G-formula (formula 12 of Cortese and Andersen^16^) to evaluate the individual risk under the observed HC. The risk of depression had no one started HC methods was derived similarly, except that the transition hazard from nonexposed to exposed was set to 0. The risk difference between these 2 scenarios is referred to as the HC-associated depression risk in the postpartum period in the investigated population.

Exploratively, we investigated the association between postpartum timing of COC initiation and depression risk (we were not able to reliably test this for the other types due to limited sample sizes). As both time since delivery^17^ and time since COC initiation^1^ may influence the depression risk, we included both time scales in a flexible parametric multistate model (eMethods 2 in Supplement 1 provides details).^18^

To assess the proportionality hazards assumption for the exposure, we considered a Cox model with a time-varying coefficient for the exposure using the cox.aalen function of the timereg package.^19^ The exposure showing violation of the proportional hazards assumption was modeled with a flexible parametric survival model via the stpm2 function of the rstpm2 package to display the HR as a function of time.^20^ To assess the sensitivity of the estimates to the proportional hazards assumption on the covariates, we used a test based on Schoenfeld residuals to identify possible violation of the proportional hazards assumption. The baseline hazard of the Cox regression model was stratified on the variables showing evidence for nonproportionality.

We conducted 5 sensitivity analyses. First, to account for other potential confounders, such as cultural differences in health care use, lifestyle factors, and parental socioeconomic status, we adjusted for immigration status (immigrant or descendant of an immigrant), smoking status, body mass index (<18.5, 18.5-24, 25-29, ≥30 [calculated as weight in kilograms divided by height in meters squared]), highest obtained educational level of the parents, and the number of different HC types previously used, as this may indicate treatment attempts of medical indications for HC use. Due to considerable missing information about body mass index (30.4%), the parents’ educational level (7.8%), and smoking status (7.9%), we imputed missing values based on the listed covariates, exposure, and outcome with the MICE package^21^ where binary variables were imputed with logistic regression and categorical data with polytomous logistic regression with 10 iterations and 5 imputations. Second, we redefined the PNOC group to only include LNG-IUS to reduce the risk of confounding by indication. Third, a 28-day delay in HC exposure was used to account for possible delays in HC initiation. Fourth, we right-censored women if they became pregnant within the follow-up time to mitigate any potential confounding related to a subsequent pregnancy. Fifth, we stratified based on prior mental disorder, as studies suggest that history of a mental disorder moderates findings on HC use and depression risk.^22,23^

All analyses were conducted using R version 4.2.2 (R Foundation for Statistical Computing).^24^ The analysis plan was preregistered.^25^ Data analysis was performed from March 20, 2023, to January 17, 2025.

## Results

Of 610 038 first-time mothers included in the study, 248 274 (40.7%) started using HC (mean [SD] age, 27.6 [4.3] years for HC users vs 29.6 [4.8] years for nonusers), of whom 143 751 (23.6%) initiated COC, 5465 (0.9%) CNOC, 66 612 (10.9%) POP, and 32 446 (5.3%) PNOC within the first year after delivery. Forty-eight (0.008%) women died within 12 months after delivery and 3287 (0.54%) were lost to follow-up due to emigration. The mean (SD) follow-up time was 11.7 (1.4) months. Their characteristics and clinical profiles are reported in Table 1. The timing of initiation of the different types is illustrated by cumulative incidence curves in eFigure 3 in Supplement 1. Approximately 50% of those who initiated POPs filled their first prescription between 7 and 10 weeks post partum. The mean (SD) follow-up exposure time for HC was 7.7 (2.9) months; for COC, 7.0 (3.0) months; CNOC, 7.4 (3.0) months; POP, 8.8 (2.2) months; and PNOC, 8.0 (2.5) months.

**Table 1.** Characteristics and Clinical Profiles of Cohorts

Profile	Participants by HC use, No. (%)						
	Nonusers	HC users	COC users	CNOC users	POP users	PNOC users	
						All users	IUS users
Total	361 764 (59.3)	248 274 (40.7)	143 751 (23.6)	5465 (0.9)	66 612 (10.9)	32 446 (5.3)	29 864 (4.9)
Maternal age, y							
<20	6429 (1.8)	8426 (3.4)	6108 (4.2)	323 (5.9)	1039 (1.6)	956 (2.9)	480 (1.6)
20-29	194 036 (53.6)	171 993 (69.3)	104 171 (72.5)	3720 (68.1)	44 203 (66.4)	19 899 (61.3)	18 122 (60.7)
≥30	161 299 (44.6)	67 855 (27.3)	33 472 (23.3)	1422 (26.0)	21 370 (32.1)	11 591 (35.7)	11 262 (37.7)
BMI^a^							
<18.5	10 983 (3.0)	7079 (2.9)	3632 (2.5)	214 (3.9)	2167 (3.3)	1066 (3.3)	891 (3.0)
18.5-24	162 580 (44.9)	108 611 (43.7)	49 853 (34.7)	3052 (55.8)	36 281 (54.5)	19 425 (59.9)	18 341 (61.4)
25-29	48 914 (13.5)	38 059 (15.3)	19 003 (13.2)	1063 (19.5)	11 403 (17.1)	6590 (20.3)	6093 (20.4)
≥30	26 394 (7.3)	21 729 (8.8)	11 577 (8.1)	581 (10.6)	5904 (8.9)	3667 (11.3)	3246 (10.9)
Educational level^b^							
Below high school	47 340 (13.1)	42 448 (17.1)	29 817 (20.7)	1227 (22.5)	7395 (11.1)	4009 (12.4)	2554 (8.6)
High school/vocational education	123 022 (34.0)	103 969 (41.9)	67 357 (46.9)	2120 (38.8)	24 920 (37.4)	9572 (29.5)	8816 (29.5)
Bachelor’s degree or above	187 427 (51.8)	100 777 (40.6)	45 951 (32.0)	2075 (38.0)	34 007 (51.1)	18 744 (57.8)	18 412 (61.7)
Highest educational level of parents^c^							
Below high school	50 562 (14.0)	40 258 (16.2)	28 407 (19.8)	759 (13.9)	8059 (12.1)	3033 (9.3)	2361 (7.9)
High school/vocational education	145 299 (40.2)	120 518 (48.5)	73 409 (51.1)	2583 (47.3)	30 619 (46.0)	13 907 (42.9)	12 622 (42.3)
Bachelor’s degree or above	129 051 (35.7)	77 059 (31.0)	37 021 (25.8)	1814 (33.2)	24 340 (36.5)	13 884 (42.8)	13 422 (44.9)
Familial disposition for mental disorder	36 859 (10.2)	27 869 (11.2)	14 254 (9.9)	759 (13.9)	8214 (12.3)	4642 (14.3)	4040 (13.5)
History of mental disorder	65 720 (18.2)	44 382 (17.9)	22 213 (15.5)	1275 (23.3)	12 980 (19.5)	7914 (24.4)	7015 (23.5)
Immigrant or descendant of immigrant	55 378 (15.3)	18 606 (7.5)	9055 (6.3)	514 (9.4)	6334 (9.5)	2703 (8.3)	2334 (7.8)
Civil status	140 647 (38.9)	70 553 (28.4)	40 693 (28.3)	1433 (26.2)	19.549 (29.3)	8878 (27.4)	8467 (28.4)
Smoking status^d^	40 006 (11.1)	39 414 (15.9)	27 479 (19.1)	1024 (18.7)	7342 (11.0)	3569 (11.0)	2620 (8.8)
Medical indication for HC	19 062 (5.3)	10 161 (4.1)	5145 (3.6)	259 (4.7)	2944 (4.4)	1813 (5.6)	1689 (5.7)
IVF treatment	64 073 (17.7)	14 260 (5.7)	7587 (5.3)	292 (5.3)	4234 (6.4)	2147 (6.6)	2074 (6.9)
Prediabetes or pregestational diabetes	11 634 (3.2)	6677 (2.7)	3447 (2.4)	133 (2.4)	1895 (2.8)	1202 (3.7)	1079 (3.6)
Eclampsia/preeclampsia	15 895 (4.4)	11 376 (4.6)	6484 (4.5)	225 (4.1)	3058 (4.6)	1609 (5.0)	1467 (4.9)
Preterm birth^e^	21 206 (5.9)	15 608 (6.3)	9513 (6.6)	308 (5.6)	3984 (6.0)	1803 (5.6)	1605 (5.4)
Instrument-assisted delivery	121 217 (33.5)	83 345 (33.6)	49 471 (34.4)	1860 (34.0)	22 173 (33.3)	9841 (30.3)	9019 (30.2)
Previous HC use							
None	73 605 (20.3)	13 400 (5.4)	8904 (6.2)	194 (3.5)	2841 (4.3)	1461 (4.5)	1163 (3.9)
1 Type	244 562 (67.6)	200 763 (80.9)	127 183 (88.5)	2507 (45.9)	50 103 (75.2)	20 970 (64.6)	19 632 (65.7)
2 Types	37 117 (10.3)	28 929 (11.7)	6810 (4.7)	2363 (43.2)	11 674 (17.5)	8082 (24.9)	7345 (24.6)
≥3 Types	6480 (1.8)	5182 (2.1)	854 (0.6)	401 (7.3)	1994 (3.0)	1933 (6.0)	1724 (5.8)

Abbreviations: BMI, body mass index (calculated as weight in kilograms divided by height in meters squared); CNOC, combined nonoral contraceptive; COC, combined oral contraceptive; HC, hormonal contraceptive; IUS, intrauterine system; IVF, in vitro fertilization; PNOC, progestogen-only nonoral contraceptive; POP, progestogen-only pill.

^a^ Information missing on mothers initiating HC: HC, 29.3%; COC, 41.5%; CNOC, 10.2%; POP, 16.3%; PNOC, 5.2%; and IUS, 4.3%. Information missing on 31.2% of those not initiating HC.

^b^ Information missing on mothers initiating HC: HC, 0.4%; COC, 0.4%; CNOC, 0.8%; POP, 0.4%; PNOC, 0.4%; and IUS, 0.3%. Information missing on 1.1% of those not initiating HC.

^c^ Information missing on mothers initiating HC: HC, 4.2%; COC, 3.4%; CNOC, 5.7%; POP, 5.4%; PNOC, 5.0%; and IUS, 4.9%. Information missing on 10.2% of those not initiating HC.

^d^ Information missing on mothers initiating HC: HC, 6.9%; COC, 9.4%; CNOC, 2.5%; POP, 4.0%; PNOC, 2.8%; and IUS, 2.8%. Information missing on 8.6% of those not initiating HC.

^e^ Information missing on mothers initiating HC: HC, 0.7%; COC, 1.0%; CNOC, 0.5%; POP, 0.4%; PNOC, 0.3%; and IUS, 0.3%. Information missing on 1.3% of those not initiating HC.

Within 12 months after delivery, 9251 of the first-time mothers (1.5%) developed depression. The crude incidence rate of depression was 21 per 1000 person-years for mothers exposed to HCs and 14 per 1000 person-years for nonexposed mothers, resulting in an adjusted HR (ie, the instantaneous risk) of 1.49 (95% CI, 1.42-1.56) (Table 2).

**Table 2.** Hazard Ratios for Depression in the Postpartum Period for HC-Exposed Compared With Nonexposed Mothers

Exposure	Person-years	HC exposure time, mean (SD), mo^a^	No. of events	AHR (95% CI)^b^	AHR (95% CI)^c^	AHR (95% CI)^d^
Nonexposed	434 620	NA	5980	1 [Reference]	1 [Reference]	1 [Reference]
HC exposed	156 928	7.7 (2.9)	3271	1.53 (1.46-1.61)	1.49 (1.42-1.56)	1.45 (1.38-1.52)
COC	83 246	7.0 (3.0)	2157	1.83 (1.73-1.93)	1.72 (1.63-1.82)	1.67 (1.58-1.76)
CNOC	3334	7.4 (3.0)	121	2.20 (1.84-2.64)	1.97 (1.64-2.36)	1.82 (1.52-2.19)
POP^e^	48 886	8.8 (2.2)	640	NA	NA	NA
PNOC	21 462	8.0 (2.5)	353	1.42 (1.27-1.58)	1.40 (1.25-1.56)	1.35 (1.21-1.51)
IUS	19 722	8.0 (2.5)	272	1.22 (1.08-1.38)	1.27 (1.12-1.44)	1.24 (1.09-1.40)

Abbreviations: AHR, adjusted hazard ratio; CNOC, combined nonoral contraceptive; COC, combined oral contraceptive; HC, hormonal contraceptive; IUS, intrauterine system; NA, not applicable; PNOC, progestogen-only nonoral contraceptive; POP, progestogen-only pill.

^a^ Restricted mean follow-up time under HC exposure (restricted to 12 months) with use of reverse Kaplan-Meier estimator.

^b^ Adjusted for age and calendar year.

^c^ Adjusted for age, calendar year, educational level, civil status, history of mental disorder, parental disposition for mental disorders, medical indications for HC use, in vitro fertilization (IVF) treatment, preterm birth, instrument-assisted or cesarean delivery, preeclampsia/eclampsia, pregestational or gestational diabetes. Missing values were handled as a separate group for educational level and preterm delivery status.

^d^ Adjusted for age, calendar year, educational level, civil status, history of mental disorder, parental disposition for mental disorders, medical indications for HC use, IVF treatment, preterm birth, instrument-assisted or cesarean delivery, preeclampsia/eclampsia, pregestational or gestational diabetes, immigration status, parental educational level, smoking status, body mass index, and number of HC types previously used. Missing values were handled by the use of multiple imputations for educational level, parental education, smoking status, body mass index, and preterm delivery status.

^e^ Hazard ratios for POPs violated the nonproportional hazard assumption; hence, the time-varying HR is shown in eFigure 4 in Supplement 1.

When stratified on HC type, the adjusted HR was 1.72 (95% CI, 1.63-1.82) for COC, 1.97 (95% CI, 1.64-2.36) for CNOC, 1.40 (95% CI, 1.25-1.56) for PNOC, and specifically for LNG-IUS it was 1.27 (95% CI, 1.12-1.44). As POP exposure showed time-varying HR, the HR for POP is represented as a function of time in eFigure 4 in Supplement 1, which shows an HR less than 1 early post partum, which subsequently increased across the postpartum period to be significantly increased from about 8 months post partum. For mothers younger than age 20 years, HC initiation post partum was associated with depression, with an HR of 1.62 (95% CI, 1.37-1.93), which was 1.55 (95% CI, 1.46-1.64) for mothers aged 20 to 29 years and 1.35 (95% CI, 1.24-1.47) for mothers aged 30 years or older (eTable 2 in Supplement 1). Adjustment for immigration and smoking status, body mass index, number of HC types previously used, and highest obtained educational level of the parents led to very similar estimated HRs (Table 2; eTable 2 in Supplement 1).

When the proportional hazards assumption was relaxed for covariates, for which there was evidence indicating violation of the proportional hazard, the estimates did not change remarkably, except the estimated HR for the mothers younger than 20 years that was considerably lower (1.34; 95% CI, 1.12-1.62; instead of 1.62; 95% CI, 1.37-1.93) (eFigure 5 in Supplement 1). Sensitivity analyses showed similar results (eFigure 6 in Supplement 1), but the HR was 1.63 (95% CI, 1.53-1.73) for women with no prior mental disorder (n = 499 936) and 1.32 (95% CI, 1.23-1.41) for women with a prior mental disorder (n = 110 102), and the ratio between the 2 was 1.24 (95% CI, 1.13-1.35).

**Figure 1.**
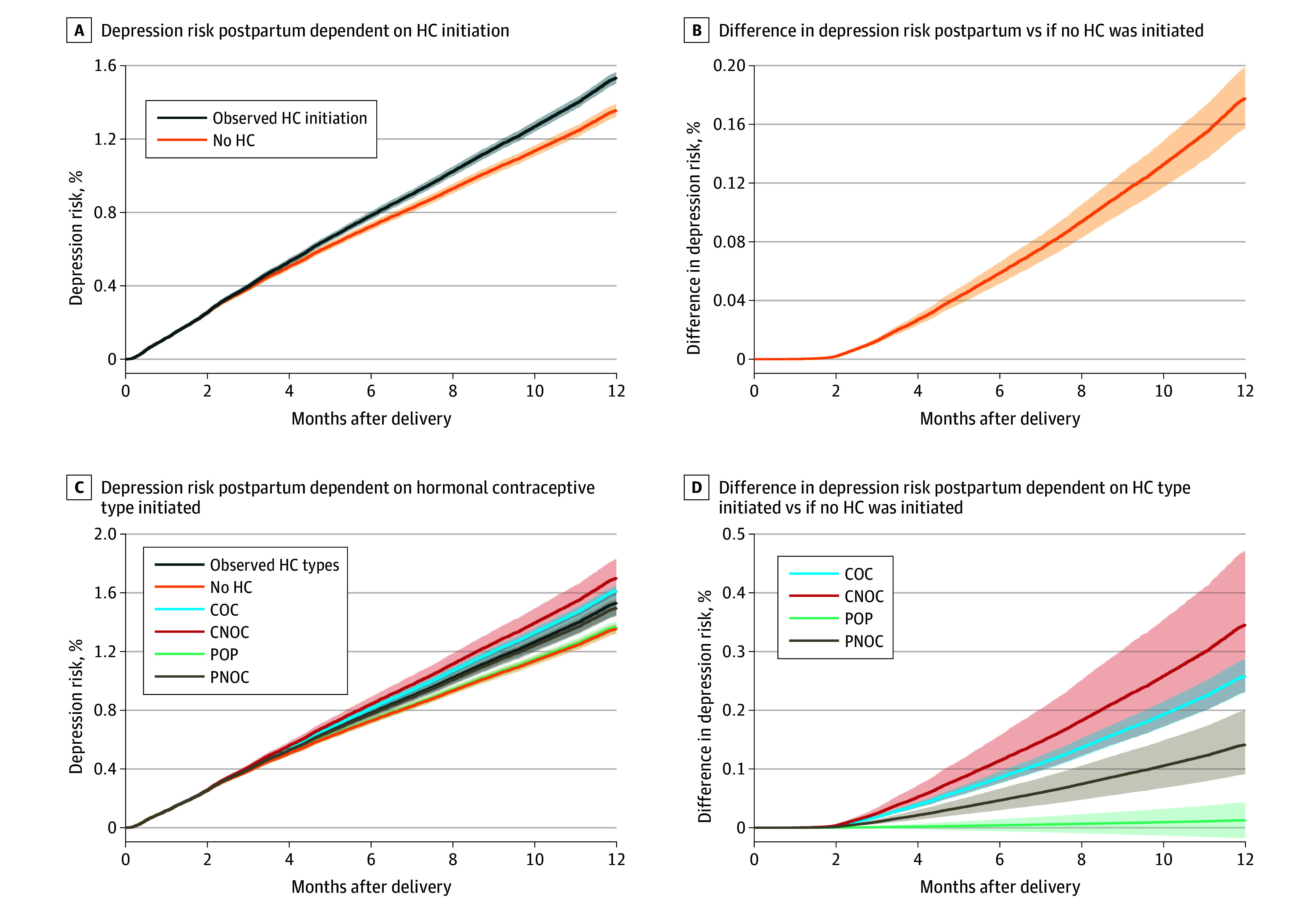
Risks of Depression at 12 Months Post Partum A, Average absolute risk of depression within 12 months from delivery under scenario 0 (observed initiation of hormonal contraceptive [HC] post partum) vs scenario 1 (nobody had initiated HC). B, Average absolute risk difference within 12 months post partum between these 2 scenarios. C, Average absolute risk of depression within 12 months from delivery for the scenarios where all mothers who were observed to initiate HC initiated only combined oral contraceptive (COC) (scenario 2), combined nonoral contraceptive (CNOC) (scenario 3), progestogen-only pill (POP) (scenario 4), or progestogen-only nonoral contraceptive (PNOC) (scenario 5), with the observed transition intensity observed for each type vs if nobody had initiated HC. D, Average absolute risk difference within 12 months post partum contrasting scenario 0 to scenarios 2 to 5. Shaded areas indicate 95% CIs.

The average absolute risk of depression at 12 months post partum for the study population under the observed HC use was 1.54% (95% CI, 1.50%-1.57%) (Table 3). In comparison, in the hypothetical scenario, if no one had initiated HC, the estimated average risk was 1.36% (95% CI, 1.32%-1.39%) (Figure 1A), resulting in a risk difference of 0.18% (95% CI, 0.16%-0.20%) (Figure 1B). Had all those who initiated HC started the COC method, the estimated average risk would have been 1.62% (95% CI, 1.58%-1.65%); with CNOC, 1.70% (95% CI, 1.58%-1.83%); POP, 1.37% (95% CI, 1.33%-1.41%); and PNOC, 1.50% (95% CI, 1.44%-1.56%), with a risk of 1.45% (95% CI, 1.40%-1.52%) specifically for LNG-IUS (Table 3, Figure 1C-D). When the proportional hazards assumption was relaxed, the estimated risks were similar, but the CIs were typically wider (eTable 3 and eFigure 7 in Supplement 1).

**Table 3.** Twelve-Month Average Absolute Risk of Depression in the Postpartum Period

Scenario	% (95% CI)^a^	
	Absolute risk	Absolute risk difference
No HC initiation^b^	1.36 (1.32 to 1.39)	1 [Reference]
Observed HC initiation	1.54 (1.50 to 1.57)	0.18 (0.16 to 0.20)
All initiating HC start on COC^c^	1.62 (1.58 to 1.65)	0.26 (0.23 to 0.29)
All initiating HC start on CNOC^c^	1.70 (1.58 to 1.83)	0.35 (0.23 to 0.47)
All initiating HC start on POP^c^	1.37 (1.33 to 1.41)	0.01 (−0.02 to 0.04)
All initiating HC start on PNOC^c^	1.50 (1.44 to 1.56)	0.14 (0.09 to 0.20)
All initiating HC start on IUS^c^	1.45 (1.40 to 1.52)	0.10 (0.04 to 0.16)

Abbreviations: CNOC, combined nonoral contraceptive; COC, combined oral contraceptive; HC, hormonal contraceptive; IUS, intrauterine system; PNOC, progestogen-only nonoral contraceptive; POP, progestogen-only pill.

^a^ Adjusted for age, calendar year, educational level, civil status, history of mental disorder; parental disposition for mental disorders, medical indications for HC use, in vitro fertilization treatment, preterm birth, instrument-assisted or cesarean delivery, preeclampsia/eclampsia, pregestational or gestational diabetes. Missing values were handled by imputing to separate groups for educational level and preterm delivery status.

^b^ A hypothetical scenario was obtained by setting the transition hazard from nonexposed to HC exposed to 0.

^c^ A hypothetical scenario was obtained by setting the transition hazard from nonexposed to COC, CNOC, POP, PNOC, and IUS by adding the transition hazard for each type and by setting the transition hazard to 0 for the rest of the types.

**Figure 2.**
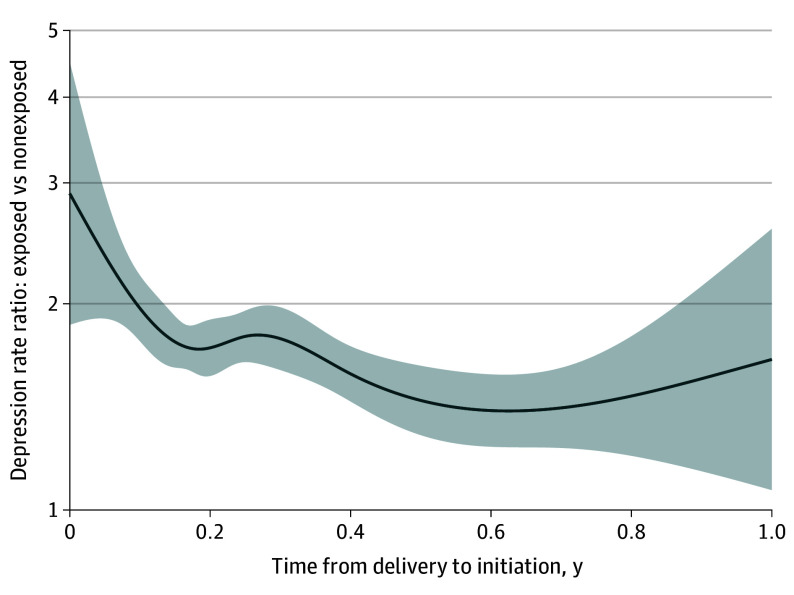
Postpartum Timing of Combined Oral Contraceptive Initiation and Depression Rate Ratio Compared With Nonexposed Mothers Ratio between an exposed mother who starts a t-year (x-axis) and a nonexposed mother in a model with a spline on the time to initiation without including time since initiation as a time scale. When assuming a linear relationship instead of a spline, the association between depression rate ratio in exposed vs nonexposed mothers and time to initiation was estimated to 0.61 (95% CI, 0.48-0.79). This means that the rate ratio decreases with time to initiation, but as illustrated by the graph, the rate ratio remains above 1 for first-time mothers who initiated treatment across all 12 months post partum compared with the nonexposed; however, after 7 months, it was estimated with high uncertainty due to the short remaining follow-up time.

Exploratively, we found no evidence of an association between time since initiation of COC and the depression rate (eFigure 8 in Supplement 1); hence, the association between the timing of initiation and depression rate was modeled without including time since initiation as a time scale. Early postpartum COC initiation was associated with a higher rate of depression, which gradually decreased during the first 7 months compared with no use (Figure 2; eFigure 9 in Supplement 1). The rate ratio remained above 1 during the whole period. Likelihood-ratio tests showed evidence of a negative linear association between time to COC initiation and depression rate with a rate ratio (per year after delivery) of 0.61 (95% CI, 0.48-0.79).

## Discussion

This study observed that HC initiation post partum was associated with an increased risk of depression 1 year post partum across all age groups. This was more pronounced for women with no prior mental disorder. The risk was increased for COCs, CNOCs, and PNOCs, but not for POP exposure.

Previous research has shown conflicting results.^4^ An observational study based on military health care system data found an increased risk of antidepressant use among women using subdermal implants and vaginal rings, no risk among COC or LNG-IUS users, and a reduced risk among POP users.^26^ As Roberts and Hansen^26^ only followed up POP users for 4.3 months on average (in contrast to 8.8 months in our study), they may have missed essential follow-up time; we observed a time-varying POP effect across 12 months post partum, which may reflect a delay between filling a prescription and actual time of initiation. Mothers in both studies filled a POP prescription shortly after delivery; however, other data have reported that despite having an early postpartum POP prescription, only about half of new mothers use them 3 and 6 months post partum, either because they have not started the medications yet or because of early discontinuation.^27^ In Denmark, as many as 1 of 4 mothers filled only 1 POP prescription.^6^ The lower associated risk in the early postpartum phase may also be explained by selection bias; progestogen-only contraceptives are recommended over combined hormonal contraceptives while breastfeeding due to a putative negative impact on lactation^12^; hence, mothers who filled a POP prescription early post partum might be overrepresented by breastfeeding mothers. Accordingly, 13% of POP users switch to COCs within 12 months post partum, which may reflect a switch after termination of breastfeeding.^6^

The association between timing of COC initiation and depression risk may indicate that the early postpartum period is a window of higher susceptibility; the interplay between the abrupt hormonal changes and psychological stressors may moderate the risk related to HC use. Alternatively, it may also be explained by selection bias, as those who initiate COCs early after delivery may represent a selected group of nonbreastfeeding mothers or mothers with a medical indication for choosing COCs over the recommended progestogen-only products.

Two studies with a more precise track of time of initiation have supported an association between postpartum progestogen exposure and the development of depressive symptoms.^28,29^ A double-blind, randomized placebo-controlled trial showed an increased risk of depressive symptoms in women allocated to a single depot injection compared with placebo at 6 weeks, but not 3 months post partum,^28^ and a randomized study found more depressive symptoms reported at 1 and 3 months post partum in women allocated to depot injections compared with women allocated to copper IUS.^29^

The higher depression risk associated with HC exposure in women without compared with women with a prior mental disorder is supported by previous observational studies.^22,23,30^ However, this is in contrast to a randomized study where prior or ongoing mental disorders were a risk factor for HC-induced mood lability.^31^ This discrepancy may be explained by a healthy user bias in the observational studies, that is, women with a prior mental disorder may be less likely to be prescribed HCs due to reported mood-related adverse effects. Alternatively, it may reflect that HC exposure plays a less prominent role relative to other risk factors in the development of clinical depression in a high-risk group. Nevertheless, it shows that HC-associated depression is seen despite no prior mental disorder, which is also supported by a prospective study reporting postpartum mood symptoms associated with HC exposure irrespective of psychiatric symptoms during pregnancy.^32^

In contrast to studies outside the postpartum period,^1,2,3^ our study found no conclusive pattern of higher risk associated with HC use among the younger compared with older women after stratifying on covariates showing evidence of time-varying effects. It has been hypothesized that a younger brain under development, that is, in adolescence, may be more susceptible to exogenous hormones,^22^ but such a difference in susceptibility may attenuate due to the structural and functional brain changes happening in relation to pregnancy and childbirth.^33^

The higher risk of depression in the postpartum period associated with HC initiation highlights the importance of considering HC exposure as a link to the already heightened risk of depression in women in the postpartum period. Furthermore, providing that our explorative analysis is replicated, the timing of initiation may be important to consider since the early postpartum period may represent a relevant window of vulnerability. This should be considered especially at postpartum contraceptive counseling where a history of HC-associated mood deterioration, premenstrual dysphoric disorder, or postpartum depression may add to such risk profiling.^34,35^

### Strengths and Limitations

One strength of the study is the use of national health registers, providing a nationwide, unselected study population with information on various potential confounders. Furthermore, it involves a population of women who had all just given birth for the first time and who were advised to consider contraceptive methods at postpartum counseling. This approach may reduce some of the potential confounders related to the decision to start HC, which might be more present at other lifetime periods.

Our study also has several limitations. First, the study is observational; hence, it is not possible to infer a causal link. Second, depression is not always the indication for antidepressant use, which may lead to a misclassification bias; however, as many as 80% of antidepressants are prescribed for depression during pregnancy,^36^ and if such a misclassification is expected to be nondifferential, it would bias the results toward the null.^37^ Third, the day a prescription was filled may not mirror the day of initiation or whether HCs were actually used post partum, potentially leading to a misclassification that could bias toward the null. Fourth, the findings may also be attenuated by a healthy user bias due to women not starting HCs post partum because of previous adverse experiences. Fifth, our findings may not extend to milder cases of depression, which do not require medication or specialist referral and therefore go undetected. Undetected cases are a potential source of bias if the frequency of detection is exposure dependent beyond the covariates included in the Cox proportional hazards regression model.

## Conclusions

In this cohort study of 610 038 first-time mothers, HC initiation was associated with an increased risk of depression in the postpartum period. This was observed for COCs, CNOCs, and PNOCs, but was inconclusive for POPs. We observed no age trend on the association between HC use and depression. These findings raise the issue of whether the incidence of depression post partum may be inflated by routine HC initiation, which is important information to convey at postpartum contraceptive counseling.

## Notes

Supplement 1. **eMethods 1.** The Multistate Markov Cox Models **eMethods 2.** Flexible Parametric Multistate Model With Multiple Timescale **eFigure 1.** Study Population Flowchart **eFigure 2.** Multistate Models **eFigure 3**. Cumulative Incidence Curves for Postpartum Initiation of Hormonal Contraception **eFigure 4.** Time-Varying Hazard and Hazard Ratio for POP Exposure **eFigure 5.** Hazard Ratio of Depression Compared to Non-Users Dependent on Stratification of Covariates Showing Evidence of Violating the Proportional Hazard Assumption **eFigure 6.** Sensitivity Analyses **eFigure 7.** Risk Curves for Depression Postpartum When Proportional Hazard Assumptions Were Relaxed for the Transition Intensity From the Exposed and the Non-Exposed State to Depression State in the Multistate Markov Cox Model **eFigure 8**. Depression Rate in Relation to Time Since Delivery, Time Since Initiation and Time From Delivery to Initiation of Combined Oral Contraception **eFigure 9**. Depression Rate at Different Timings of Initiation of Combined Oral Contraception Postpartum **eTable 1.** Overview of Registers, Variables, and Codes **eTable 2.** Instantaneous Risk of Depression Postpartum Stratified on Age Groups **eTable 3.** 12-Month Average Absolute Risk of Depression in the Postpartum Period When the Proportional Hazard Assumption Was Relaxed

Supplement 2.
Data Sharing Statement
