# Supplementary material for: Postpartum Hormonal Contraceptive Use and Risk of Depression
Source: JAMA Netw Open. 2025 Mar 31;8(3):e252474. doi: 10.1001/jamanetworkopen.2025.2474 (PMC11959440; doi:10.1001/jamanetworkopen.2025.2474)
Supplement: Supplement 2. — Data Sharing Statement [file jamanetwopen-e252474-s002.pdf]

Data Sharing Statement

Larsen. Postpartum Hormonal Contraceptive Use and Risk of Depression. *JAMA Netw Open*. Published March 31, 2025. doi:10.1001/jamanetworkopen.2025.2474

Data
Data available: No

Additional Information
Explanation for why data not available: Danish national health register data cannot be distributed, but access to the data can be granted by the appropriate authorities.
